# The Future Public Health Workforce in a Changing World: A Conceptual Framework for a European–Israeli Knowledge Transfer Project

**DOI:** 10.3390/ijerph18179265

**Published:** 2021-09-02

**Authors:** Osnat Bashkin, Keren Dopelt, Zohar Mor, Lore Leighton, Robert Otok, Mariusz Duplaga, Fiona MacLeod, Jascha De Nooijer, Yehuda Neumark, Stephanie Paillard-Borg, Theodore Tulchinsky, Shira Zelber-Sagi, Nadav Davidovitch

**Affiliations:** 1 Department of Public Health, Ashkelon Academic College, Ben Tzvi 12, Ashkelon 78211, Israel; dopelt@bgu.ac.il (K.D.); zohar.mor@moh.gov.il (Z.M.); tulchinskyted@hotmail.com (T.T.); 2 Department of Health Policy and Management, School of Public Health, Faculty of Health Sciences, Ben Gurion University of the Negev, P.O. Box 653, Beer Sheva 8410501, Israel; nadavd@bgu.ac.il; 3 The Association of Schools of Public Health in the European Region (ASPHER), Avenue de Tervueren 153, 1150 Brussels, Belgium; lore.leighton@aspher.org (L.L.); robert.otok@aspher.org (R.O.); 4 Department of Health Promotion and e-Health, Institute of Public Health, Faculty of Health Sciences, Jagiellonian University Medical College, ul. Skawińska 8, 31-066 Kraków, Poland; mariusz.duplaga@uj.edu.pl; 5 School of Public Health, University College Cork, T12 XF62 Cork, Ireland; f.macleod@ucc.ie; 6 School of Health Professions Education, Faculty of Health, Medicine and Life Sciences, Maastricht University, P.O. Box 616, 6200 MD Maastricht, The Netherlands; j.denooijer@maastrichtuniversity.nl; 7 Braun School of Public Health and Community Medicine, Hebrew University-Hadassah, Jerusalem 91120, Israel; yehudan@ekmd.huji.ac.il; 8 Department of Health Sciences, The Swedish Red Cross University College (SRCUC), P.O. Box 1059, 14121 Huddinge, Sweden; pais@rkh.se; 9 Faculty of Social Welfare and Health Sciences, School of Public Health, University of Haifa, 199 Aba Khoushy Ave. Mount Carmel, Haifa 3498838, Israel; szelber-s@univ.haifa.ac.il; 10 The Israeli Association of Public Health Physicians (IPAPH), Israeli Medical Association, P.O. Box 3566, Ramat Gan 5213604, Israel

**Keywords:** public health workforce, public health education, Israel public health services, capacity building, Erasmus+

## Abstract

Health services quality and sustainability rely mainly on a qualified workforce. Adequately trained public health personnel protect and promote health, avert health disparities, and allow rapid response to health emergencies. Evaluations of the healthcare workforce typically focus on physicians and nurses in curative medical venues. Few have evaluated public health workforce capacity building or sought to identify gaps between the academic training of public health employees and the needs of the healthcare organizations in which they are employed. This project report describes the conceptual framework of “Sharing European Educational Experience in Public Health for Israel (SEEEPHI): harmonization, employability, leadership, and outreach”—a multinational Erasmus+ Capacity Building in Higher Education funded project. By sharing European educational experience and knowledge, the project aims to enhance professionalism and strengthen leadership aspects of the public health workforce in Israel to meet the needs of employers and the country. The project’s work packages, each jointly led by an Israeli and European institution, include field qualification analysis, mapping public health academic training programs, workforce adaptation, and building leadership capacity. In the era of global health changes, it is crucial to assess the capacity building of a well-qualified and competent workforce that enables providing good health services, reaching out to minorities, preventing health inequalities, and confronting emerging health challenges. We anticipate that the methods developed and the lessons learned within the Israeli context will be adaptable and adoptable by other countries through local and cultural adjustments.

## 1. Introduction

“At the heart of each and every health system, the workforce is central to advancing health.” [1]. This project report describes the conceptual framework of an international project which aims to transfer lessons learned in the education and training of the public health workforce in Europe to address the needs of employers in Israel. At the same time, the tools and framework developed for this project will be adaptable for use in other socio-cultural contexts, and the results will be relevant for other countries in Europe and beyond.

### 1.1. The Healthcare System in Israel

The central structure of the healthcare system in Israel is based upon the national health insurance program established in 1995 through the enactment of the National Health Insurance Law [2]. Under this law, which views health as a fundamental right, every Israeli citizen is entitled to a “basket” of healthcare services provided through four public healthcare insurers (health funds) and financed through compulsory health insurance (~5% of income) [3].

Preventive medicine and other public health functions are organized and provided by the Public Health Services (PHS) of the Ministry of Health through local health bureaus. In addition, the health funds have also recognized the public health, economic, and societal value of prevention and health promotion in reducing the burden of communicable and non-communicable diseases and trauma-related conditions. Health promotion activities, mainly on the municipal level, take place in many settings, including schools, workplaces, and community centers. By emphasizing public health (PH) and disease prevention, Israel has made remarkable progress in recent decades in reducing birth and infant mortality rates and improving health quality measures such as life expectancy [4], and also recently in its robust COVID-19 response [5]. Despite these achievements, Israel faces ongoing challenges related to social determinants of health such as poverty and inequities between population groups (e.g., Ethiopian immigrants, Arabs, Bedouins, and ultra-orthodox Jews as compared to the middle and upper economic Jewish population, especially in major urban centers) [6,7,8]. Emerging challenges include climate change [9], food insecurity [10], and new globally transmitted diseases such as COVID-19. These complicated challenges require a further innovative capacity building that will address the global health changes that are taking place in different local contexts. The efficiency of the public healthcare system relies on an infrastructure of well-trained, multidisciplinary professionals who are capably involved with designing and implementing public health and health promotion programs to meet these challenges.

### 1.2. Public Health Workforce in Israel

The Ministry of Health (MOH) manages the PHS, comprising a national headquarters, operating regional and district offices, and various field units. These units are staffed by PH physicians, PH nurses, environmental engineers, and other PH-related professions, such as dieticians, health promoters, and epidemiologists [2]. In 1994, the Ministry of Health established the Israel Center for Disease Control (ICDC), which plays essential roles in data collection, monitoring, and analysis of communicable and non-communicable diseases, as well as conducting surveys related to a variety of health behaviors. The field of health promotion is active through the Department of Health Promotion in the MOH, which aims to enable the population to increase control over their own health and to improve it, working with different sectors involved in health promotion, especially in settings such as schools, workplaces, and different communities. In addition, the health funds are increasingly involved in health education, health promotion, and disease prevention programs. Regrettably, unlike the basket for clinical services and treatments, a “health promotion basket” is yet to be implemented. Moreover, the contents of a national basket of health promotion and prevention programs and services remain to be defined [2].

In Israel, as elsewhere, the public health workforce (PHW) is challenging to define, classify, and enumerate due to the absence of professional licensure or certification. Furthermore, there is no consensus on the designation of a “public health practitioner”, and central registries of PH professionals (apart from public health physicians and nurses) do not exist [11,12]. The lack of professional categorization and recognition at the regulatory level detracts from the attractiveness of being a member of the PHW [13].

### 1.3. Public Health Education in Israel

Five Israeli academic institutions provide training programs in PH; four universities offer Master of Public Health (MPH) programs: The Ben Gurion University of the Negev, The Hebrew University of Jerusalem, University of Haifa, and Tel Aviv University. In 2015, an undergraduate PH program was established at the Ashkelon Academic College [14]. The Israeli Association of Public Health Physicians (IPAPH), which is a medical society in the Israeli Medical Association (IMA), is developing the syllabus and standards of PH physicians’ training in collaboration with the Israeli PHS [2]. Public health nursing in Israel is one of the specialized post-graduate fields for nurses. These nurses are working mainly in Mother and Child Health Centers and as nurse epidemiologists. Nurse epidemiologists have an active role in the prevention and control of communicable diseases, including carrying out epidemiological investigations, identifying sources of infection, and methods of infection. They are also involved in health education and notification of certain diseases like measles, diphtheria, tetanus, etc., to health authorities. In addition, they teach and supervise other workers in surveillance activities [15]. Other public health professionals include dentists, veterinarians, and sanitary engineers, with options for specialized MPH tracks designated for them. Other professions within public health tracks include those in fields such as health promotion, environmental epidemiology, biostatistics, health communication, public health economics, and public health law.

### 1.4. Assessment of Public Health Academic Programs: Findings from the Israeli Council for Higher Education Evaluation

In May 2017, the Israeli Council for Higher Education (CHE) evaluated several PH programs in higher education institutions (HEIs) [16]. CHE met with HEI stakeholders, including management, faculty, staff, students, and alumni. Based on the evaluations, CHE published recommendations both at the individual, institutional level and overall, for Israeli academic PH programs (CHE reports are available in English at https://che.org.il/wp-content/uploads/2018/07/PH-and-PHM-General-Evaluation-Report.pdf (accessed on 12 July 2021)):

Following up the CHE reports, Israeli ASPHER members met with ASPHER and selected European Union ASPHER partners to consider how Israeli PH programs might benefit from best practices in Europe to improve their public health education delivery and better align the PHW in Israel to meet the requirements of employers. These discussions led to a proposal to learn from European-tested solutions and tools described in this concept paper. The proposal was submitted to the Erasmus+ program for Capacity Building in Higher Education (CBHE, KA2 Cooperation for innovation and the exchange of good practices) and awarded funding for a project period of three years (January 2021–January 2024). This concept paper describes the “Sharing European Educational Experience in Public Health for Israel (SEEEPHI): harmonization, employability, leadership, and outreach” project.

#### 1.4.1. Defining Core Elements in Public Health Education in Israel

The European Agency for Public Health Education Accreditation (APHEA) and the Council on Education for Public Health (CEPH) in the US recognize five traditional core areas of PH: (1) biostatistics, (2) epidemiology, (3) environmental health, (4) health policy and management, and (5) social and behavioral sciences. The CHE Committee observed and concluded that education in PH in Israel lacks agreement on the core areas of PH and how and to what extent those core elements should be included in educational programs. Therefore, it was recommended to establish working groups and subcommittees in each of the five core areas of PH to define core elements and educational standards [16].

#### 1.4.2. Developing Practice-Oriented Public Health Programs

To ensure the delivery of essential health services, the professional PHW requires up-to-date theoretical knowledge alongside practical skills in a broad range of core competencies, especially considering the continuous evolution of the field [17,18]. The CHE Committee identified the need to balance traditional academic PH education with professional, practice-oriented training. It was noted that MPH graduates should have a professional identity comparable to other professional healthcare-related degrees. Schools of PH in Israel have recognized the need to prepare all PH students to better meet the needs of employers to confront challenges in PH. Strategies to accomplish this goal include adopting experiential, problem-based learning (PBL) and teaching leadership skills at all education and training levels [19], as is done in many European PH schools and programs [20].

#### 1.4.3. Content Assessment and Learning Outcomes Development

The Bologna Working Group on Qualifications developed the concept of “Learning Outcomes” to specify expectations of what a student should learn from a particular course and its curriculum for programs in Europe [21]. The CHE Committee observed that most PH programs in Israel have not fully embraced the concept of Learning Outcomes and recommended that all programs adopt processes for developing Learning Outcomes and regular assessment of course and curriculum content to ensure that graduates possess the skills and knowledge necessary to be effective PH professionals. It was also recognized that clarification and harmonization of Learning Outcomes across degree programs would allow students to move more efficiently through their educational trajectory and prevent gaps and overlaps in curriculum content. Competencies assessments in use in Europe, particularly ASPHER’s European Core Competencies List for the Public Health Professional [22], would aid in ensuring students meet the desired Learning Outcomes for PH at each step of their education.

## 2. Sharing European Educational Experience in PH for Israel (SEEEPHI): Harmonization, Employability, Leadership, and Outreach

As suggested by its name, the purpose of the SEEEPHI project described herein is to engage European HEI partners to share education and training approaches and to harmonize the PH educational system in order to maximize the employability of graduates in Israel. Moreover, the project aims to enhance professionalism and strengthen the leadership aspects of the PHW in Israel. To gain deeper insights, the project includes outreach to employers, together with advocacy of PH legislation and policies within Israel. The skills required of the PHW are changing, and the schools of PH in Israel are recommended to make changes in their programs that some of their European counterparts have already undertaken.

Health systems vary significantly between and within countries, including within the EU. Among the challenges faced by all of Europe’s healthcare systems, and Israel’s as well, are those that revolve around the disadvantage of groups, such as ethnic minorities, due to limited access to health services. The health problems faced by these groups are often not easily identified in health system assessment reports and, furthermore, the gathering and analysis of data vary greatly between EU countries. Clinical interventions, access to treatment, or preventive care also differ considerably. However, the EU has good potential, through its diversity, to strengthen the use of transparent healthcare assessment and delivery.

European HEIs have examined solutions that may be offered to respond to the needs identified by the CHE [23]. ASPHER, in partnership with other European-level organizations such as the World Health Organization (WHO) Regional Office for Europe, has developed a tool for assessing the qualifications required by employers in the PHW [22]. This tool is designed to be adaptable for use across the European region’s diverse national, professional, and educational contexts and can, thus, also be tailored to Israel’s unique setting.

This project allows Israel the opportunity to benefit from methods developed in European HEIs to train new faculty and instructors in PBL and leadership training. Unlike Israel, where bachelor-level training in PH is just beginning to develop, European HEIs have long-standing PH training for bachelor students. They can share relevant experience on academic progression, employment, and career opportunities in the PHW.

### 2.1. Conceptual Framework of the SEEEPHI Project

The project brings together a consortium of schools of PH in Europe and Israel. The project’s main goal is the enhancement of the public health workforce in Israel through sharing European educational experience, including harmonization, employability, leadership, and outreach.

Under the aim of the project, the SEEEPHI project has defined the following work packages (WP):

#### 2.1.1. Detailed Analysis of Field Qualifications Content to Understand Different Professional Roles in the Israeli Public Health System

To enhance the PHW in Israel, the current professional roles and functions that comprise the national PH system, and those foreseen to be required in the coming decade, must be identified and redefined. To ensure adequate implementation of standards and functions, explicit descriptors of competencies are needed to identify potential skills gaps and inform the development of training and educational programs to match job market needs. Mapping field qualification expectations through surveys and key stakeholder interviews will enable the comparison of competencies required by employer organizations and those provided by HEI educational programs (see Section 2.1.2). Developing a comprehensive profile of employers’ PHW needs—both present and anticipated—will allow HEIs in Israel to tailor academic programs to meet the real-world conditions that await students upon graduation and strengthen ties between HEIs, communities, and employers.

The WHO-ASPHER Competency Framework for the Public Health Workforce in the European Region [24] provides a framework to evaluate the competencies needed by PH employers at three workforce levels—entry, competent, and expert. The multi-level approach of the framework will allow the Israeli HEIs to understand the qualifications required of PH students.

#### 2.1.2. Mapping the Competency Profiles of the Israeli Schools and Programs of Public Health to Guide Harmonization between Public Health Education and Practice

In parallel to describing the professional toolbox needed by the PH system and potential employers, the SEEEPHI project will map the competencies of bachelor-level and master-level PH programs offered at HEIs in Israel. ASPHER’s European Core Competencies List for the Public Health Professional [22] will be applied to accomplish this objective. This mapping exercise will highlight curriculum commonalities and differences and serve as the basis for discussions about the harmonization of educational programs across HEIs. The competencies-mapping results will be compared with those of the employer survey to identify gaps between the contents of the PH graduate’s toolbox and the toolbox required at the three workforce levels.

#### 2.1.3. Development and Launching of a Dynamic Online Interface to Enable Public Health Education/Training—Practice/Workforce Collaborations, Supporting Employability and Continuing Professional Development in the Israeli Public Health System

This work package will include developing an online platform for ongoing interactions between the PH system, HEIs, employers, students, alumni, faculty, and PH professionals. It will provide information about the internships and employment, as well as opportunities for individual career development guidance. ASPHER’s European Public Health Reference Framework (EPHRF) will serve as a model for developing the Public Health Israel EPHRF platform, in addition to Practical Placement Schemes each semester and annual Career Fair events.

#### 2.1.4. Building Leadership Capacity via Cutting-Edge Training in the Israeli Public Health Schools and Programs, including Peer-to-Peer and Train-the-Trainers Offerings

Improvement in the education of PH students will be achieved, in part, through the training of faculty and staff at Israeli HEIs in practical PBL and leadership training techniques. This must include methods to evaluate students’ achieved learning outcomes appropriately. In Israel, planning is underway to build a Leadership Training Academy in PH, and this project will support this effort accordingly.

The Leadership for Public Health in Europe (LEPHIE) training program was designed in recognition that courses offered by schools of PH often failed to meet the needs and expectations of the PHW. LEPHIE addresses the need for studies in leadership and critical thinking. The experiences and lessons from LEPHIE and the teaching methodologies used in European HEIs can be transferred to the Public Health Israel Leadership Academy under development, which will then serve to sustain training activities locally.

#### 2.1.5. Stakeholder Engagement to Secure Key Outreach (Community, Inter-Professional, Cross-Sectoral) Needed to Sustain the Proposed Solutions

Stakeholder engagement with employers and the community must be assured. HEIs in PH must also build partnerships with key professional groups needed to promote the integration of PH graduates across the broader PHW in Israel, encourage awareness for prioritization in resource allocation for PH, and advance professional recognition of the Israeli PHW. A campaign will be developed to raise awareness of PH and its roles, expanding on the ongoing “This is Public Health” campaign [25] to feature a new “I am Public Health” component in order to showcase roles and functions filled by members of the PHW. Visibility for the campaign will be provided online and at various events and conferences in Israel. Campaign efforts to promote recognition and strengthen the professional identity of the PHW will be supported by the establishment of a PHI Registry.

**Figure 1:**
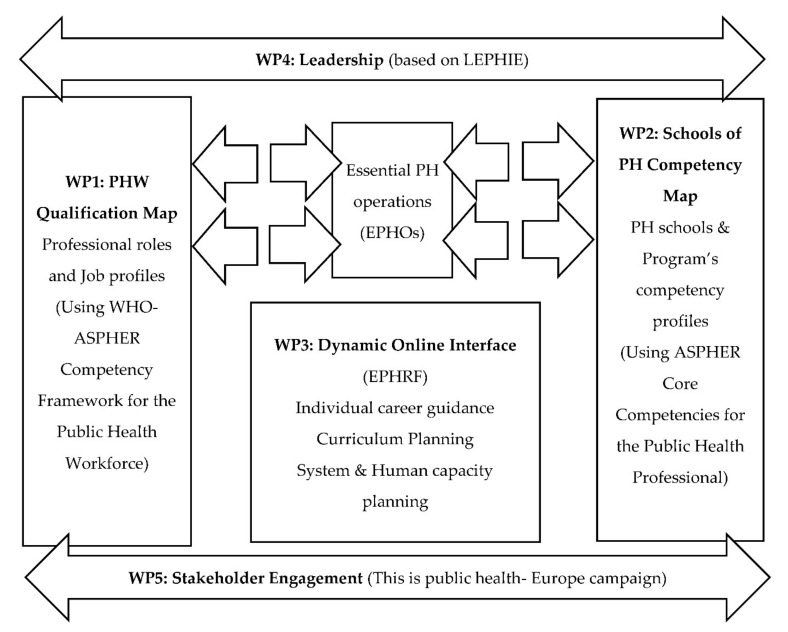
Work Packages of SEEEPHI.

Figure 1 summarizes the five work packages’ content and the project framework.

### 2.2. Expected Outcomes and Practical Implications

The project will address the challenges described and the CHE recommendations by understanding the needs of employers and communities that the PHW serves, as well as the subsequent development of measures to ensure that schools and programs of PH can train students in the field qualifications needed to meet the expectations of the job market.

The Israeli HEIs and the broader Israeli PHW will be able to benefit from voluntary incorporation of European tools, best practice, and methodologies, including learning and teaching methodologies and pedagogical approaches; new forms of practical training schemes and study of real-life cases in business and industry; university–enterprise cooperation; guidance, counseling, and coaching methods and tools; and tools and strategies for professionalization and professional development.

Transferring knowledge will also allow acquisition of insights into the conditions under which best practices were developed and implemented, and the use of the same approach to develop and implement best practices in Israel while considering the local context.

The project has been designed to address implementation challenges common to similar projects, such as inertia, lack of trust among all stakeholders, unrealistic expectations within a stressed system, and sustainability of changes.

Another aspect of this project is the role of leadership and inclusion of different parts of society. The project will take local communities into account at each step by: including local organizations in its outreach and determination of field qualifications; building the findings of these assessments into the recommendations for harmonization and development of PH degrees and continuing education programs; and seeking to place students in practicums, internships, and employment in local communities and, thereby, to strengthen the PHW servicing these communities. This will encourage both the exchange of knowledge and student recruitment to educate future PH leaders from within minority groups and to sustain contacts within the minority community organizations in order to continually build and react to their changing needs.

## 3. Conclusions

The SEEEPHI project brings together a consortium of schools of PH in Europe representing historically diverse PH systems from eastern, Nordic, continental, and non-continental (the British Isles) regional contexts. This rich diversity of collective experience of the European HEIs offers Israeli schools the opportunity to benefit from various solutions in order to find the best fit to transform education and training approaches and harmonize the educational system to maximize the employability of their graduates. Israel is a young society with a diverse population from many ethnic, religious, cultural, and social backgrounds. Each group has its own health needs and, therefore, cultural and structural sensitivity is required within Israel as well.

The project described in the current paper comes at a time of evaluation and national self-reflection on programs of PH education. The critical role of the PHW in responding to, and eventually recovering from, the COVID-19 pandemic emphasizes the need to assess the PHW. In the era of global health changes, it is crucial to adequately adapt academic training to workforce needs and build leadership in PH. Capacity building of a well-qualified and competent workforce will enable the provision of responsive and high-quality health services, reaching out to minorities, addressing health inequalities, and confronting emerging health challenges. The model developed through SEEEPHI may serve other countries facing similar challenges, adapted to their needs, priorities, history, structure, and local context.

## Data Availability

No new data were created or analyzed in this study. Data sharing is not applicable to this article.
